# Transactions of the Chicago Society of Physicians and Surgeons, November 23, 1874

**Published:** 1874-12-01

**Authors:** Ralph E. Starkweather


### TRANSACTIONS OF THE CHICAGO SOCIETY OF PHYSICIANS AND SURGEONS.

### REGULAR MEETING, NOVEMBER 23, 1874.

*Reported by Ralph E. Starkweather, M.D.*

DR. JOHN BARTLETT, President, in the chair.

Prof. DeLafontaine gave to the Society his views upon the hydrate of chloral in its action, together with details of experiments made by him and by Dr. C. P. Simon, upon rabbits, cats and sheep, by the hypodermic injections of chloral.

Prof. DeLafontaine thought that the hydrate of chloral diminished the available oxygen of the blood by poisoning the red discs by the carbonic oxide gas set free in the action of chloral. That a person frequently taking chloral hydrate suffers from its effects as he would from frequent venesection. In poisoning by chloral, the blood taken from the jugular vein before death is red as carmine; after death, for some time, it will not coagulate, and the condition is like that of one who dies from suffocation. The test by the spectroscope for the carbonic oxide was then given. If the blood is healthy there will be the two dark bands of absorption, yellow and green; if the blood has been poisoned by the carbonic oxide the bands will likewise be the same; but when treated by the hydro-sulphide of ammonium the two dark bands coalesce in the normal blood, but there is no such effect whatever in that which is poisoned. The chloral changes the form of the red discs; their shape is lost and they become elongated, shrivelled and rugose. Frequent use of chloral will ultimately ruin the health, as much so as the constant use of morphine.

Dr. Simon, in relating the experiments he had made, remarked that he was not quite of the same opinion as to the dangers of the hydrate of chloral as expressed by his friend Prof. DeLafontaine. The subject of anæsthesia and chloral and the experiments made by Dr. Simon and Prof. DeLafontaine were then discussed by the Society.

Dr. Owens reported a case which he had previously reported as recovered, in which the patient has since died. It was an operation for the removal of a colloid multilocular ovarian tumor. The patient died from septicæmia. A chart of the pulse, respiration and temperature accompanied the paper, and also a report by Dr. Danforth on the microscopical examination of the tumor and its contents.

Dr. Hollister read an elaborate article upon the subject of catalepsy, illustrating the same by details of a case of rare interest now under treatment at the Mercy Hospital. The full details of this case will be published in an early number of the EXAMINER.

